# Aortic Valve Leaflet Avulsion After Transcatheter Aortic Valvuloplasty: A Case Series and Literature Review

**DOI:** 10.1016/j.jaccas.2025.105232

**Published:** 2025-09-24

**Authors:** Ammar Abusulb, Robert Cubeddu, Brian Solomon, Travis Howard, Dee Dee Wang, Luis H. Paz Rois, Mazen Albaghdadi

**Affiliations:** Cardiovascular Medicine, Rooney Heart Institute–Naples Community Hospital, Naples, Florida, USA

**Keywords:** aortic valve, aortic valve leaflet avulsion, BAV, TAVR

## Abstract

**Background:**

Aortic valve leaflet avulsion (AVLA) is recognized as a life-threatening adverse event that significantly affects patient hemodynamics, often leading to additional intervention.

**Case Summary:**

We report 2 cases from our institution of AVLA after transcatheter aortic valve replacement (TAVR). The patients had a highly mobile mass on the aortic annulus and the left ventricular outflow tract, respectively.

**Discussion:**

In both cases, a multidisciplinary team discussion resulted in the decision to monitor the patients for 48 hours for neurovascular changes while on therapeutic anticoagulation, and both patients were discharged on anticoagulants and low-dose aspirin. Their hospital courses were uneventful, and normally functional aortic valve bioprostheses were noted on follow-up.

**Beyond the Guidelines:**

The literature on AVLA after TAVR remains limited. The current treatment approaches for AVLA are primarily invasive, including surgical repair of the valve or snaring of avulsed structures.

**Take-Home Message:**

Conservative management may be appropriate in post-TAVR AVLA cases without ischemic or embolic manifestation.

## Visual Summary

**Figure:**
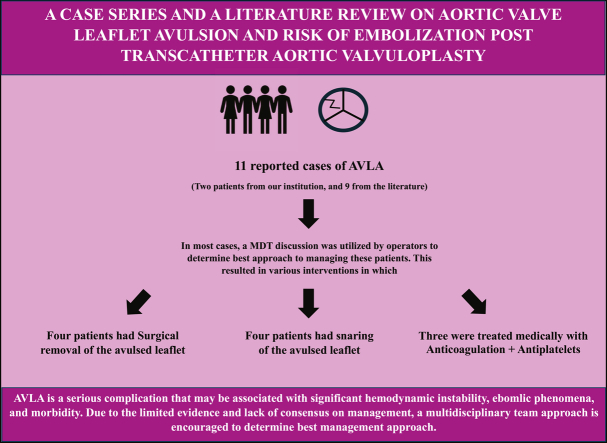

**Visual Summary:**
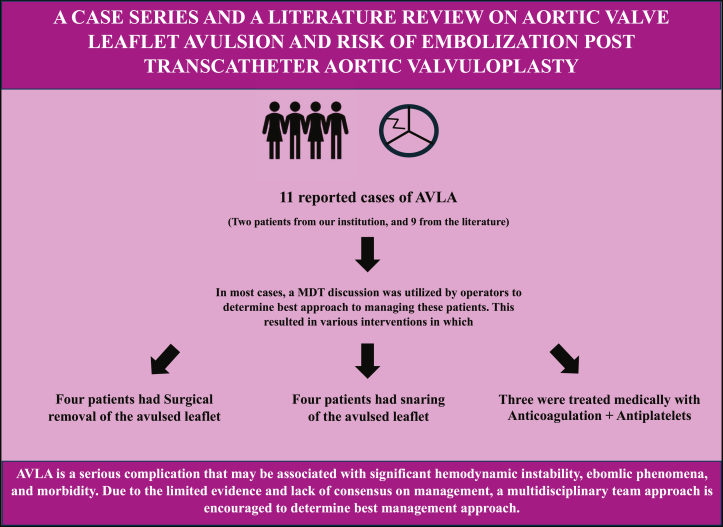
Summary of Reported AVLA Case Series From 2014 Until the Present Time After multidisciplinary team discussions, 5 of the 12 cases were managed with surgical removal of the avulsed valve, 4 were managed with snaring of the avulsed leaflet, and 3 were managed medically. AVLA = aortic valve leaflet avulsion; MDT = multidisciplinary team.

Transcatheter aortic valve replacement (TAVR) is now emerging as the dominant therapy for patients with symptomatic aortic valve stenosis. AVLA has been recognized as a life-threatening adverse event with a significant impact on patient hemodynamics, often leading to significant morbidity or the need for more invasive intervention^1^, ^2^, ^3^ Because of the low reported incidence of AVLA, there are limited data on periprocedural predictors, outcomes, and management.

**Take-Home Messages**

• AVLA after TAVR is a serious complication that may be associated with significant hemodynamic instability, embolic phenomena, and morbidity.
• Given the increase in TAVR volume worldwide, AVLA will be more commonly encountered, and a multidisciplinary heart team approach is critical in light of the limited evidence and lack of consensus on the management of this potentially life-threatening complication.

We report 2 cases of AVLA from our institution, with our approach to diagnosis and management. We also summarize 9 previously reported cases of AVLA,^4^, ^5^, ^6^, ^7^, ^8^, ^9^, ^10^, ^11^, ^12^ including patient characteristics, management, and outcomes.

## Patient 1

A 91-year-old woman with a history of systolic heart failure, paroxysmal atrial fibrillation, and chronic kidney disease developed progressive, symptomatic severe aortic valve stenosis and was deemed appropriate for TAVR.

**Figure 1:**
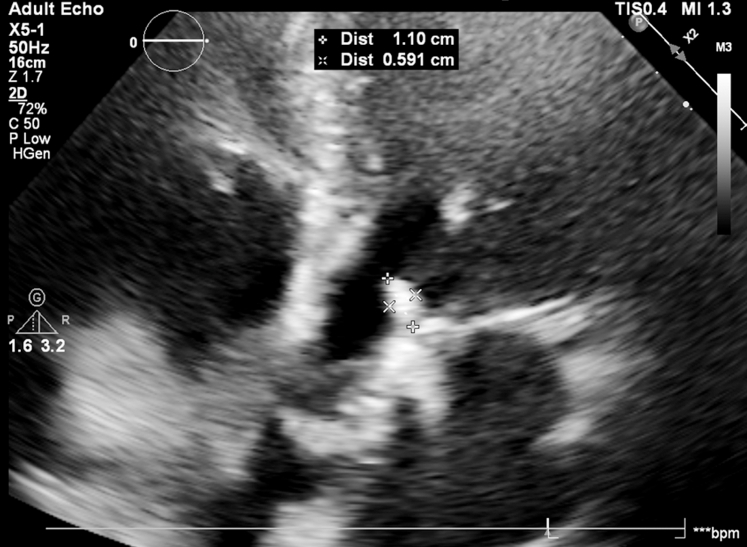
Frozen Image From the TTE of Patient 1 This image demonstrates a new echodensity that was not seen or appreciated on the patient's prior TTE. The echodensity was measured at 1.1 × 0.591 cm. TTE = transthoracic echocardiogram.

She had reduced left ventricular ejection fraction, with a calculated valve area of 0.8 cm^2^. A dobutamine stress echo confirmed the presence of low-flow, low-gradient severe aortic stenosis (Video 1, Video 2, Video 3, Video 4, Video 5). After transfemoral TAVR, echocardiogram demonstrated no significant residual gradient, and no paravalvular or valvular regurgitation. No balloon aortic valvuloplasty (BAV) was performed either before or after TAVR. However, a linear mobile echodensity was observed on transthoracic echocardiogram (TTE) in the left ventricular outflow tract (LVOT), and aortography demonstrated no evidence of coronary obstruction (Figure 1, Video 6, Video 7, Video 8). The possibility of thrombus versus avulsion of the native aortic valve leaflet was discussed. After a multidisciplinary team discussion, the consensus opinion was to manage the avulsed leaflets with anticoagulation and serial echocardiography in the absence of hemodynamic instability, embolic events, or coronary ischemia. The patient was treated with unfractionated heparin and was monitored in the cardiovascular intensive care unit with frequent neurological examinations.

The patient did not experience any change clinically while being monitored in the intensive care unit. A repeat TTE demonstrated that the previously observed mobile echodensity in the LVOT, thought to be an AVLA, was no longer present and was presumed to have embolized without clinical consequence. She was discharged home after 48 hours of post-TAVR monitoring and was instructed to resume her home Eliquis, which she was previously taking in the setting of her atrial fibrillation.

The patient was seen in the clinic 1 week after discharge and was doing well with no complaints. A repeat TTE 1 month post-TAVR showed no changes in her left ventricular ejection fraction or the implanted bioprosthetic aortic valve.

## Patient 2

An 81-year-old woman with a history of hypertension was referred for evaluation of progressive dyspnea on exertion and near-syncope.

Her TTE showed a preserved left ventricular ejection fraction with severe critical aortic valve stenosis (Video 9, Video 10, Video 11, Video 12, Video 13). TTE after transfemoral TAVR showed the implanted valve in an excellent position, with mild to moderate paravalvular regurgitation successfully treated with postdilation single inflation. A repeat TTE showed the resolution of the paravalvular regurgitation, however, a highly mobile mass was noted in the LVOT (Video 14, Video 15, Video 16, Video 17, Video 18). The mass was further explored on transesophageal echocardiogram (TEE), which showed it appearing to be attached to the aortic annulus and mobile within the LVOT region but without evidence of LVOT obstruction. The possibility of thrombus versus avulsion of the native aortic leaflet was discussed.

The TAVR team elected to manage the AVLA conservatively given no evidence of aortic insufficiency, mitral regurgitation, coronary obstruction, or aortography and because of the lack of any hemodynamic or ischemic impact of the mobile mass. The patient did not have any signs or symptoms of an embolic event. Repeat TTE showed no significant change, the previously identified LVOT mass no longer visualized, and the aortic bioprosthesis demonstrated stable position and function. She was discharged after 72 hours from the TAVR procedure on Eliquis and low-dose aspirin.

The patient was seen in the clinic 1 week after discharge and was doing well with no complaints. A repeat TTE 1 month post-TAVR showed no significant changes.

## Previously Reported Cases

### Methods

Major databases, including PubMed, Embase, and Google Scholar, were searched for English-language articles and case reports published from inception until August 2024 using the keywords “aortic valve,” “avulsion,” and “TAVR.” The reference lists of relevant case reports were also reviewed to identify any additional case reports. We included any reported cases that involved AVLA and either native or bioprosthetic valves. Ethical approval was not required according to our institutional guidelines.

### Results

We identified 9 case reports on AVLA after TAVR that were published between January 2014 and July 2024.^4^, ^5^, ^6^, ^7^, ^8^, ^9^, ^10^, ^11^, ^12^ The average age of the patients was 80.6 years, and 7 of the 9 patients (77.8%) were male. The avulsed leaflets occurred in the native valve in 7 patients and in the bioprosthetic valve after surgical aortic valve replacement in 2 patients; the information was not reported in 1 case. BAV was performed in 6 (66.7%) of the cases and was not performed in 2 of the cases. Four of the patients developed hemodynamic instability, which was mainly hypotension, which did not occur in 2 of the cases and was not reported in the remaining cases (n = 3). See Table 1 for further details.

**Table 1:** Reported Cases of Aortic Valve Avulsion Between 2014 and February 2024

Published Year	Age (y)/ Sex	Existing Valve Pre-TAVR	Aortic Valve Area (cm^2^)	Avulsed Coronary Cusp	Preprocedure Balloon Valvuloplasty	Periprocedural Hemodynamic Instability
2014^11^	83/male	Native	0.552	2 with a calcified nodule in the middle	Performed with 23 mm × 4 cm valvuloplasty balloon (Edwards Lifesciences)	Hypotension upon discontinuation of rapid pacing
2016^12^	86/female	Native	0.4	Right	Performed with 20-mm Edwards balloon	None
2016^7^	82/male	Bioprosthetic	NR	NR	NR	None
2017^8^	78/male	Native	0.7	Left	Performed with 18 × 40 mm Tyshak balloon	NR
2020^6^	77/male	Bioprosthetic	0.6	NR	Not performed	Yes
2020^9^	78/male	Bioprosthetic	0.576	Left	Performed with postdeployment balloon dilatation with28 × 40 mm Z-MED balloon	NR
2023^4^	92/female	Native	0.67	Right	Not performed	Hypotensive predeployment requiring ECMO support
2024^10^	76/male	Native	0.56	NR	Performed with compliant 14-mm balloon	NR
2024^5^	78/male	Native	0.52	Left	Performed with a predilated18-mm Z-MED balloon	Yes, severe hypotension postvalvuloplasty

ECMO = extracorporeal membrane oxygenation; NR = not reported; TAVR = transcatheter aortic valve replacement.

Evidence of embolization was seen in 2 of the cases, but only 1 embolic event occurred in all 9 cases; in this patient, the leaflet embolized to the left lower extremity, leading to vascular insufficiency, and an open exploration of the femoral artery and embolectomy were performed. In addressing the avulsed valve leaflets, the management plan was surgical removal in 4 (44.4%) of the cases, 4 cases (44.4%) were snared, and 1 case (11.1%) was treated medically with anticoagulation and dual antiplatelet therapy for 3 months. All patients were discharged from the hospital post-TAVR, with a maximum hospital length of stay of 10 days. All patients with outcomes data (n = 6) were asymptomatic on outpatient follow-up. See Table 2 for further details.

**Table 2:** Reported Cases of Aortic Valve Avulsion Between 2014 and February 2024

Published Year	Evidence of embolization/Embolic event	Valve Type	Valve Size, mm	Management Plan	Case Outcome	Follow Up
2014^11^	Did not occur	Edwards Sapien	26	Palmaz stent to temporarily trap the mobile tissue structure, then snared the mass	Well-positioned valve on cardiac imaging	No reported issues at 2-mo follow-up
2016^12^	Did not occur	Edwards Sapien	23	3 mo of dual antiplatelet agents and warfarin	No events during hospitalization and discharged after 5 d	Repeat TEE at 3 mo showed a mobile echodensity, unchanged in from procedural TEE
2016^7^	Did not occur; both the valve and the leaflet were retrieved together	CoreValve (Evolut-R)	26	Leaflet was retrieved with the first attempt, reinsertion of a second valve was done after	NR	NR
2017^8^	Did not occur	Sapien XT valve	29	Anticoagulation (Xarelto) and 18-mm to 30-mm EN-snare retrieval 30 d later	Normal function valve postprocedure	No reported issues at 1-mo follow-up
2020^6^	Embolized causing lower extremity vascular insufficiency	CoreValve (Evolut-R)	26	LLE angiography and surgical retrieval	Discharged on postoperative day 2	No reported issues at 1-mo follow-up
2020^9^	Did not occur	CoreValve (Evolut-R)	34	Post-TAVR valve avulsion, patient had SAVR with 27-mm Avalus valve + CABG to PDA	Uneventful postoperative course and discharged on postoperative day 4	NR
2023^4^	Embolized to descending aorta	Edwards Sapien 3 valve	23	Snared leaflet from the descending aorta	Normal functioning bioprosthetic valve	NR
2024^10^	Did not occur	NR	29	SAVR with a porcine bioprosthetic valve	Well-seated valve on cardiac imaging, discharged on hospital day 10 after SAVR	No reported issues on follow-up
2024^5^	Did not occur	Balloon expandable Sapien 3 prosthesis	26	Aortotomy and cusp resection	Discharged in stable condition	No reported issues at 6-mo follow-up

CABG = coronary artery bypass grafting; LLE = left lower extremity; NR = not reported; PDA = posterior descending artery; SAVR = surgical aortic valve replacement; TAVR = transcatheter aortic valve replacement; TEE = transesophageal echocardiogram.

## Discussion

AVLA is a potentially serious but fortunately rare complication after TAVR. In addition, major clinical trials do not usually mention AVLA when reporting results.^13^ Our case series and literature review highlights the uncertainties of AVLA, including predictors and outcomes. AVLA may be more frequent after BAV and in patients with prior surgical aortic valve replacement, however, the available data remain sparse.

The etiology of AVLA remains unclear based on existing reports. Proposed theories are that, while crossing the narrow aortic valve orifice during TAVR, the wire ends up entering through a leaflet, resulting in damage to the leaflet, annulus, or sometimes both.^4^^,^^6^^,^^12^ It has been suggested that performing a BAV may increase the risk of leaflet avulsion in the context of heavily degenerative native or bioprosthetic leaflets or in patients with a prior history of chest radiation.

Hemodynamic instability after TAVR be due to a range of mechanisms, including tamponade, coronary obstruction, LVOT obstruction, aortic insufficiency, embolic phenomena, vascular access site complications, and conduction disturbances. AVLA is a rare and possibly under-recognized cause of post-TAVR hemodynamic instability that may be due to coronary embolism and LVOT and/or coronary obstruction. Multiple reports have described the sudden loss of hemodynamic stability as the first manifestation of AVLA. This can also be seen in the severe aortic insufficiency after BAV due to the disruption of a native valve or surgical bioprosthetic valve. A sudden drop in blood pressure should prompt operators to further investigate the structure and integrity of the native valve before or after implantation.

Many investigators have emphasized the role of intraoperative TEE and its effectiveness in detecting the presence of both AVLA and other serious peri-TAVR complications. In many cases, TEE is invaluable in demonstrating the integrity of the native valve preimplantation and the complication when it occurs, providing additional data and guiding the management plan. However, given the possibility of detecting AVLA with TTE, the use of TTE is likely reserved for selected cases of TAVR given the benefits of a minimalist approach to TAVR.

Existing reports of AVLA remain rare, and the reported management approaches are heterogeneous. Nearly half of the cases from the literature managed AVLA with surgical retrieval in the absence of any hemodynamic or embolic manifestation. Percutaneous snaring of the AVLA may also be an option in patients with a prohibitive risk for cardiac surgery. Importantly, conservative medical management with surveillance echocardiography and anticoagulation may be reasonable in selected patients without hemodynamic or embolic manifestation. A multidisciplinary heart team discussion is key to the management of AVLA and is reported in many cases in the literature. Interventionalists, imagers, and surgeons must understand these different approaches to management, taking into account the patient's surgical risk, size of the avulsed mass, and other factors before devising their management plan.

## Conclusions

AVLA is a serious complication that may be associated with significant hemodynamic instability, embolic phenomena, and morbidity and can be managed conservatively in selected cases. To our knowledge, this is the only case series report of 2 cases of AVLA with evidence of embolization, which was treated medically with no reported complications on follow-up.

Existing literature does not support a uniform approach to AVLA, and a multidisciplinary heart team approach is critical for the management of this potentially life-threatening complication. Given the growth of TAVR volume worldwide, AVLA will be more commonly encountered. Our case series and review provides some experiential insight into the diagnosis and management of AVLA in patients undergoing TAVR.

## Funding Support and Author Disclosures

The authors have reported that they have no relationships relevant to the contents of this paper to disclose.

## Appendix

Video 1 TTE Pre-TAVR for Patient 1 Parasternal long-axis view showing heavily calcified aortic valve with limited mobility. No mobile masses or densities can be appreciated.

Video 2 TTE Pre-TAVR for Patient 1 Parasternal long-axis view with a focus on the LVOT and AV, showing heavily calcified aortic valve with limited mobility. No mobile masses or densities can be appreciated.

Video 3 TTE Pre-TAVR for Patient 1 Parasternal long-axis view with color Doppler axis view showing heavily calcified aortic valve with limited mobility. No mobile masses or densities can be appreciated. Moderate mitral regurgitation can be appreciated as well.

Video 4 TTE Pre-TAVR for Patient 1 Parasternal long-axis view showing heavily calcified aortic valve with limited mobility. No mobile masses or densities can be appreciated.

Video 5 TTE Pre-TAVR for Patient 1 Apical 3-chamber view showing heavily calcified aortic valve with limited mobility. No mobile masses or densities can be appreciated.

Video 6 TTE Post-TAVR for Patient 1 Parasternal long-axis view showing a subtle mobile echodensity that can be appreciated in some images.

Video 7 TTE Post-TAVR for Patient 1 Apical 3-chamber view showing a highly mobile echodensity that can be appreciated in the LVOT on the LV aspect.

Video 8 TTE Post-TAVR for Patient 1 Apical 3-chamber view with a focus on the LVOT that shows a highly mobile echodensity that can be appreciated in the LVOT on the LV aspect.

Video 9 TTE Pre-TAVR for Patient 2 Parasternal long-axis view showing heavily calcified aortic valve with limited mobility. No mobile masses or densities can be appreciated.

Video 10 TTE Pre-TAVR for Patient 2 Parasternal long-axis view with a focus on the LVOT and AV, showing heavily calcified aortic valve with limited mobility. No mobile masses or densities can be appreciated.

Video 11 TTE Pre-TAVR for Patient 2 Parasternal short-axis view at the level of the AV showing heavily calcified aortic valve with limited mobility. No mobile masses or densities can be appreciated.

Video 12 TTE Pre-TAVR for Patient 2 Parasternal short-axis view at the level of the AV with a focus on the AV, showing heavily calcified aortic valve with limited mobility. No mobile masses or densities can be appreciated.

Video 13 TTE Pre-TAVR for Patient 2 Apical 3-chamber view showing heavily calcified aortic valve with limited mobility. No mobile masses or densities can be appreciated.

Video 14 TTE Post-TAVR for Patient 2 Parasternal long-axis view showing a mobile echodensity in the LVOT from the LV side, which is new and was not present on prior images.

Video 15 TTE Post-TAVR for Patient 2 Parasternal long-axis view with a focus on the LVOT showing a mobile echodensity in the LVOT from the LV side, which is new and was not present on prior images.

Video 16 TTE Post-TAVR for Patient 2 Apical 4-chamber view showing a mobile echodensity in the LVOT from the LV side, which is new and was not present on prior images.

Video 17 TEE Post-TAVR for Patient 2 Midesophageal long-axis view showing a mobile echodensity in the LVOT from the LV side.

Video 18 TEE Post-TAVR for Patient 2 Color-compare, midesophageal long-axis view with color flow Doppler to outline the flow.

## References

[1] Szotek M., Drużbicki Ł., Sabatowski K., Amoroso G.R., De Schouwer K., Matusik P.T. (2023). Transcatheter aortic valve implantation and cardiac conduction abnormalities: prevalence, risk factors and management. J Clin Med.

[2] Mach M., Okutucu S., Kerbel T. (2021). Vascular complications in TAVR: incidence, clinical impact, and management. J Clin Med.

[3] Ranasinghe M.P., Peter K., McFadyen J.D. (2019). Thromboembolic and bleeding complications in transcatheter aortic valve implantation: insights on mechanisms, prophylaxis and therapy. J Clin Med.

[4] Inoue M., Shimizu M., Okamoto H. (2023). An unexpected free-floating aortic valve leaflet avulsion in the left atrium on transesophageal echocardiography during transcatheter aortic valve implantation under extracorporeal membrane oxygenation. CASE (Phila).

[5] Williams D.M., Castellano A., Phillips W. (2024). Surgical management of native aortic valve leaflet avulsion during TAVR. J Cardiothorac Surg.

[6] Kinthala S., Saththasivam P., Ankam A., Sattur S. (2020). Embolization of aortic valve leaflet during valve-in-valve transcatheter aortic valve implantation: a case report. European Heart Journal. Case Rep.

[7] Patankar G.R., Grayburn P.A., Hebeler R.F., Henry A.C., Stoler R.C. (2016). Avulsion of aortic leaflet during transcatheter aortic valve replacement. J Interv Cardiol.

[8] Sherev D., Azizi K., Azimi N.A., Van Nordheim S., Moreno-Cabral R. (2017). Delayed management of partial aortic valve avulsion after transcatheter aortic valve replacement. Ann Thora Surg.

[9] Park H., Leung Wai Sang S., Merhi W.M. (2020). Extremely early structural failure of a self-expanding transcatheter aortic valve secondary to leaflet dehiscence. JTCVS Tech.

[10] Chaturvedula S.T., Sharma A., Kiernan F. (2024). Avulsion of the native aortic valve leaflet following transcatheter aortic valve replacement: a dreaded complication. Cardiovasc Revasc Med.

[11] Zimmet J., Kaiser E., Tseng E., Shunk K. (2014). Successful percutaneous management of partial avulsion of the native aortic valve complex complicating transcatheter aortic valve replacement. J Invasive Cardiol.

[12] Smith T., Khalil R., Lasorda D. (2016). “Silent” acute native valve leaflet avulsion during transcatheter aortic valve replacement. J Cardiol Curr Res.

[13] Leon M.B., Smith C.R., Mack M. (2010). Transcatheter aortic-valve implantation for aortic stenosis in patients who cannot undergo surgery. N Eng J Med.

